# Editorial: Applications of STEM (Science, Technology, Engineering and Mathematics) Tools in Microbiology of Infectious Diseases

**DOI:** 10.3389/fmicb.2017.00215

**Published:** 2017-02-14

**Authors:** Julio Alvarez, Andres M. Perez

**Affiliations:** Department of Veterinary Population Medicine, College of Veterinary Medicine, University of MinnesotaSaint Paul, MN, USA

**Keywords:** STEM, quantitative methods, epidemiology, modeling, pathogen detection

## Introduction

Epidemiology is a discipline intended to systematically investigate, and ideally quantify, disease dynamics in populations (Perez, 2015). Epidemiological assessments may be divided into four large areas, namely, (a) identification and characterization of a pathogen, (b) development of systems for detection of cases, (c) descriptive epidemiology and quantification of disease patterns, and (d) advanced analytical methods to design intervention strategies. Briefly, there is an initial need for understanding the pathogeny of a disease and condition, which may also include experimental studies and development of new models of infection and proliferation under different conditions. Subsequently, such knowledge may be applied to support the identification of cases, which typically includes the design, evaluation, and validation of diagnostic tests. Disease may then be quantified in a population, leading to the identification of patterns and application of molecular characterization techniques to understand disease spread, and ultimately to identify factors preventing or promoting disease. Finally, those factors may be incorporated into advanced quantitative methods and epidemiological models, which are used to design and evaluate strategies aimed at preventing, controlling, or eliminating disease in the population.

Recent years have seen a dramatic increase in the application of science, technology, engineering, and mathematical (STEM) tools and approaches intended to enhance such analytical epidemiology process, with the ultimate goal of supporting disease prevention, control, and eradication. The research topic here provides an update of the current state-of-the-art scientific knowledge of the application of STEM tools to the microbiology of infectious diseases, at the various components of an integral epidemiological approach, divided into the four large components of (a) experimental studies, (b) novel diagnostic techniques, (c) epidemiological characterization, and (d) population modeling and intervention.

## Experimental studies

The increased knowledge gained in the last decades on the importance of host-pathogen and environment-pathogen interactions has provided a better understanding on the complexities associated with the design of effective therapeutic and preventive strategies against infectious agents, but also raised new questions on how to evaluate those potential new approaches in a capturing their complexity. Six papers looking into the use of STEM tools for the design of experimental models for replicating this environment and host-pathogen interactions and ultimately the assessment of alternative strategies to prevent pathogen replication were selected here. Those papers were focused on emerging and/or neglected pathogens whose control is impaired due, at least in part, to its fastidious nature (*Mycobacterium tuberculosis*) (Zhan et al.; Devasundaram et al.), its ability to form environmentally resistant structures such as biofilms and/or resist common therapeutic approaches (*Staphylococcus aureus* and *Acinetobacter baumannii*) (Atshan et al.; He et al.; Zhou et al.) or the lack of effective treatments to prevent or treat infection (*Trypanosoma brucei gambiense*) (Hamidou Soumana et al.). An *in-vitro* dormancy model was used to study genes that were overexpressed under hypoxia by *M. tuberculosis* strains recovered from disease outbreaks (as opposed to *M. tuberculosis* laboratory strains) (Devasundaram et al.). Nevertheless, complementation of evidences found *in-vitro* with results from *in-vivo* models is important because different results may be obtained in those alternative settings. As an example, murine models were used to assess antibacterial activities of a panel of 14 antimicrobial agents against multi-drug resistant *A. baumannii* (He et al.). Using an alternative approach the efficacy of a fourth generation cephalosporin (cefquinome) against planktonic and biofilm *S. aureus* cells was evaluated in another study (Zhou et al.). A mouse model was also of critical importance to mimic human latent tuberculosis infection and assess the efficacy of a fungus (*Ganoderma lucidum*) against *M. tuberculosis* H37Rv (Zhan et al.). Finally, protein and gene expression found in a pathogen (*S. aureus*) and a vector (the tsetse fly *Glossina palpalis gambiensis*, vector of *T. brucei gambiense*) were analyzed in two studies that both considered different conditions [planktonic growth vs. biofilm development for *S. aureus* (Atshan et al.) and infected vs. non-infected specimens for the *G. palpalis* (Hamidou Soumana et al.)].

## Novel diagnostic techniques

Eight papers were selected demonstrating the use of novel diagnostic techniques or the evaluation of established approached for the detection of global emergencies (Huang et al.; Li et al.; Meghdadi et al.; Pérez-Sancho et al.; Domenech et al.; Krishna et al.; Tian et al.; Yu et al.). The combined use of magnetic nanoparticles (MNPs) and giant magnetoresistance (GMR) biosensors has recently gained attention because of their potential for simultaneous, rapid and affordable detection of multiple pathogens. Monoclonal antibodies in combination with MNPs and GMR biosensors were used to detect influenza virus in swine, with a potential application to nasal samples, which are routinely collected by the swine industry worldwide (Krishna et al.). Matrix-assisted laser desorption/ionization time-of-flight (MALDI-TOF) mass spectrometry is becoming increasingly popular. In the research topic here, applications of MALDI-TOF has been demonstrated in three case-studies, including antimicrobial susceptibility testing in positive body fluid cultures collected from a hospital in Shanghai, China (Tian et al.), characterization of hypervirulent strains of *Klebsiella pneumoniae* (Huang et al.), and identification of *Streptococcus suis* isolates obtained from multiple host species (Pérez-Sancho et al.). Finally, rapid detection of emergent diseases and conditions of global importance require of novel, affordable tools that could potentially be applied at large scale in developing settings to early detect disease cases. Here, it was presented (1) a novel method using mycobacteriophage 2GFP10, which was evaluated for detecting drug resistance in clinical isolates of *Mycobacterium tuberculosis* (Yu et al.), (2) nested PCR for the detection of *Mycobacterium tuberculosis* DNA in patients with extra pulmonary tuberculosis (Meghdadi et al.), (3) a reverse transcription loop-mediated isothermal amplification (RT-LAMP) method to detect Zaire ebolavirus in Sierra Leone using the nucleoprotein gene as a target sequence (Li et al.), and a novel typing method for *Streptococcus pneumoniae* using selected surface proteins (Domenech et al.).

## Epidemiological characterization

Once the tools for detection and characterization of the pathogen of interest have been developed the challenge shifts to the interpretation of the results obtained in different populations. Four papers focusing on three bacterial pathogens (*Escherichia coli, Vibrio parahaemolyticus*, and *V. cholerae*) that exemplify these challenges and include the application of molecular biology methods were selected (Han et al.; Cabal et al.; Ghosh et al.; Han et al.). Use of molecular characterization techniques allow identification of the pathogen beyond the species level, thus offering a much higher degree of refinement that can help to understand better the epidemiology of a disease (Muellner et al., 2011) and clarify the biological relationship between different strains. In addition, when used directly on clinical samples, it may help to improve the sensitivity of the diagnostic approach used eliminating the need for isolation of the pathogen, what can be particularly useful when its concentration is low and there are other microorganisms that could outgrow it. For example, a set of specific real-time (RT) PCRs aiming at a panel of virulence genes characteristic of different *E. coli* pathotypes was used to assess their distribution and, when detected, quantify the number of copies present, in samples from clinical human cases and healthy volunteers in Spain and thus provide qualitative and quantitative information on the significance of their detection (Cabal et al.). Detection of virulence genes, coupled with other molecular (DNA sequencing, ribotyping, restriction fragment length polymorphism—RFLP—and pulsed field gel electrophoresis—PFGE) and conventional (serotyping, antimicrobial susceptibility determination) was also used for a detailed characterization of a collection of *V. cholerae* strains recovered in a 5-year period in India, revealing a considerable diversity and thus a dynamic evolution of the pathogen that must be taken into account when evaluating individual isolates (Ghosh et al.). Finally, a well-established molecular characterization technique, multi-locus sequence typing (MLST), was used to assess the genetic diversity of *V. parahaemolyticum* at the national (China) (Han et al.) and, for pandemic strains (those harboring a given set of virulence markers), global levels (Han et al.) by analyzing two collections of clinical and environmental isolates. While the first study demonstrated a high genetic diversity among strains recovered from the same country, comparable to what had been described at a global scale (Han et al.), the second evidenced the persistence of certain pandemic strains in different countries and their occasional detection in environmental samples (Han et al.).

## Population modeling and intervention

Population modeling for the characterization of spatial risk is prerequisite for the design and implementation of prevention and control interventions in a region. Three techniques (Bayesian analysis, ecological niche modeling, and phylogeography tools) have been demonstrated in this research topic (Martínez-López et al.; Alkhamis et al.; Escobar and Craft). Bayesian techniques have the potential to support the characterization of disease risk in a region because of their flexibility to adapt the coding to different data structure and variables distribution. A Bayesian multivariable logistic regression mixed model was used to assess the relation between hypothesized risk factors and African swine fever virus (ASFV) distribution in Sardinia (Italy) after the beginning of the eradication program in 1993 (Martínez-López et al.). Additionally, one of the challenges epidemiologists often face is the absence of control or population data. Ecological niche modeling is a tool that allows quantification of spatial risk using presence-only (case) data. An overview of the background, history, and conceptual framework of ecological niche modeling applied to epidemiology and public health was presented (Escobar and Craft). Finally, the use of Bayesian phylodinamic models was demonstrated using a large volume of porcine reproductive and respiratory syndrome virus (PRRSV) collected in the United States (Alkhamis et al.).

## Final remarks

The acknowledgment that health of humans, animals, and the environment is inter-connected has resulted in the establishment of the One Health approach (Perez, 2015). Cross-cutting a myriad of pathogens, host species, and settings, and under the One Health umbrella, novel STEM tools and methods have a tremendous potential to increase our ability to understand pathogens dynamics, improve effectiveness of detection, characterize the epidemiological setting in which disease spread, and, ultimately, develop effective strategies to mitigate or eliminate the impact of disease.

## Author contributions

JA and AP co-edited the Research Topic and wrote this editorial.

### Conflict of interest statement

The authors declare that the research was conducted in the absence of any commercial or financial relationships that could be construed as a potential conflict of interest.
